# Avulsion of the Lesser Trochanter Following a Shot Put Sport Session

**DOI:** 10.5811/cpcem.2016.12.32605

**Published:** 2017-03-13

**Authors:** Mohamed H. Grissa, Nasri Bzeouich, Makram Zrig, Hamdi Boubaker, Mohamed A. Msolli, Abderrazak Abid, Semir Nouira, Amanda Montgomery

**Affiliations:** * University of Monastir, Department of Emergency Medicine, Research Laboratory (LR12SP18), Monastir, Tunisia; † Fattouma Bourguiba University Hospital, Department of Emergency Medicine, Monastir, Tunisia; ‡ Fattouma Bourguiba University Hospital, Department of Orthopedics, Monastir, Tunisia; § Kern Medical, Department of Emergency Medicine, Bakersfield, California

## Abstract

Avulsion of the lesser trochanter is an uncommon injury. In children and adolescents it usually occurs as a sports injury via traumatic avulsion of the psoas major tendon. In adults, isolated fractures of the lesser trochanter are most commonly pathological due to metastatic tumor invasion of the proximal femur. This case report documents how a 14-year-old boy, who presented with an avulsion of the lesser trochanter of the proximal femur following a seemingly atraumatic shot put session at a track and field event, was diagnosed and successfully treated with a conservative approach.

## INTRODUCTION

Avulsion of the lesser trochanter during a sports event may remain undetected in a child. Emergency department (ED) diagnosis based on history and physical examination is made more difficult by the fact that this traumatic lesion is rarely associated with throwing the shot put. As this activity involves primarily the upper body, the emergency physician may not suspect an indication to order lower extremity imaging in the absence of obvious trauma.

## CASE REPORT

A 14-year-old male with no medical history reported feeling pain in his right groin region during a shot put session. The mechanism of injury was a forceful abduction and external rotation of the hip, sustained while the subject was throwing the weight (a five-kilogram steel ball).

**Image 1:**
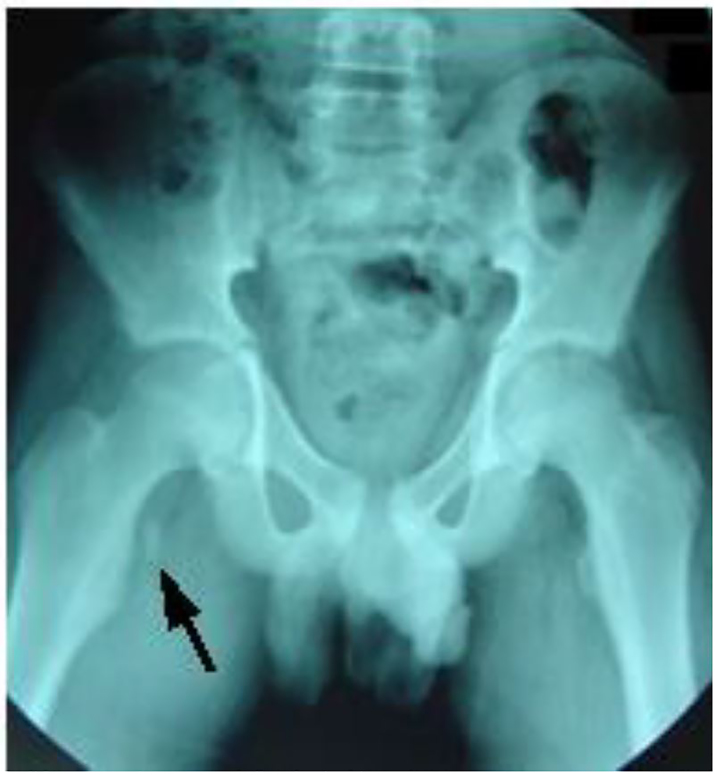
Radiograph of the pelvis of a 14-year-old patient showing an avulsion of the right lesser trochanter (arrow) compared to the left normal hip.

**Image 2:**
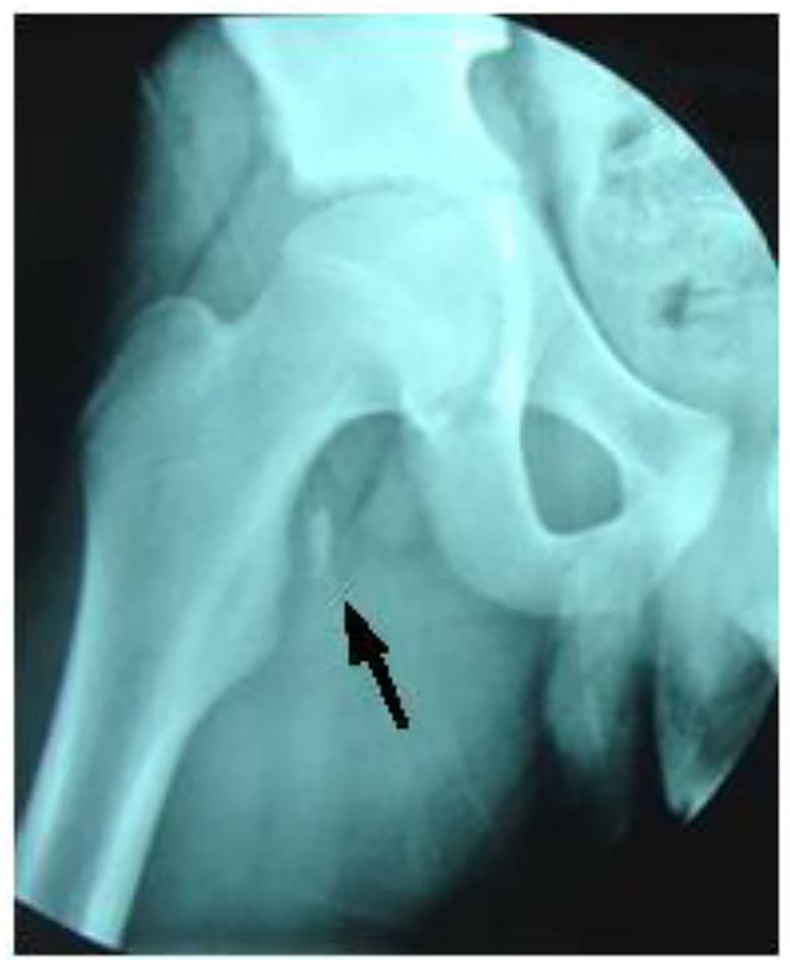
Apophyseal avulsion fracture of lesser trochanter with proximal displacement of fracture fragment (arrow).

The child was evaluated in the ED at the University Hospital of Monastir-Tunisia. Physical examination revealed a thin boy with antalgic gait of the left lower extremity. There was no obvious deformity, edema, or ecchymosis; however, he was tender on the medial aspect of the left hip. While no limitation of passive movement was appreciated, active flexion was painful. Strength was decreased, especially with flexion greater than 90 degrees. No neurological deficits were detected. Pulses were present and symmetrical in both legs. Right hip and pelvic radiographs revealed a single fragment avulsion of the lesser trochanter with 1 centimeter of cranial displacement (Images 1, 2).

A conservative therapeutic approach was implemented. The patient was treated with analgesics. He was confined to bed rest for two weeks, after which he was made non-weight bearing, on crutches, for another four weeks. After the six-week rest period, the patient was enrolled in a four-week intensive rehabilitation program designed to help him regain strength and range of motion.

## DISCUSSION

Isolated fractures of the lesser trochanter are rare injuries in childhood accounting for only 0.3 percent of proximal femur fractures.^1^ Avulsion fractures typically occur during adolescence. They are usually seen in children between the ages of 7–16 years, but most commonly occur at the age of 14.^2^

Avulsion of the lesser trochanter usually occurs from an acute injury most commonly related to sporting events.^3^ The main cause of such avulsions is forceful contraction of the iliopsoas tendon during hip flexion. The excessive stress concentrated at the site leads to a tensile failure of the apophysis of the lesser trochanter.^4^ The diagnosis may be suggested by the patient’s age and mechanism of injury. However, other causes of pediatric hip pathology, including septic arthritis, slipped capital femoral epiphysis, osteosarcoma and Perthes’ disease, must be ruled out in this age group.^2^,^5^

The most frequent presenting clinical scenario is groin pain and limp, with little external evidence of trauma. The physical examination often reveals tenderness over the medial aspect of the hip and pain with hip flexion greater than 90 degrees (Ludloff sign). The diagnosis is confirmed with radiography and is classified as a Salter-Harris Type I fracture. The avulsed fragment is most frequently displaced proximally by the pull of the iliopsoas tendon.^6^

Symptomatic treatment is recommended, with limited weight-bearing on crutches for three to four weeks and analgesia as required. Complete healing can take up to two months, and sports should be avoided during this time.^3^ Surgical intervention is indicated where a non-union or fibrous union has occurred, resulting in chronic pain with motion at the fracture site. If the avulsed fragment is displaced more than three centimeters, surgical reattachment or excision is advised.^4^ However, a study by Fernbarch and Wilkonson demonstrated that operative treatment is rarely indicated.^7^ This study looked at 20 male adolescents engaged in competitive sports. Results of those treated conservatively were comparable to open reduction and internal fixation of the fragment, regardless of the degree of displacement.

The majority of patients with this type of injury eventually become asymptomatic and are able to return to original activity levels. This is true even in the setting of persistent radiographic abnormalities.
